# Current Applications of Recycled Aggregates from Construction and Demolition: A Review

**DOI:** 10.3390/ma14071700

**Published:** 2021-03-30

**Authors:** Glaydson Simões dos Reis, Marco Quattrone, Weslei Monteiro Ambrós, Bogdan Grigore Cazacliu, Carlos Hoffmann Sampaio

**Affiliations:** 1 Université Gustave Eiffel, MAST, GPEM, F-44344 Bouguenais, France; glaydsonambiental@gmail.com (G.S.d.R.); bogdan.cazacliu@univ-eiffel.fr (B.G.C.); 2 Biomass Technology Centre, Department of Forest Biomaterials and Technology, Swedish University of Agricultural Sciences, SE-901 83 Umeå, Sweden; 3 National Institute on Advanced Eco-Efficient Cement-Based Technologies, Department of Construction Engineering, Escola Politécnica, University of São Paulo, São Paulo 05508-070, Brazil; MARCO.QUATTRONE@lme.pcc.usp.br; 4 Mineral Processing Laboratory, Federal University of Rio Grande do Sul, 9500 Bento Gonçalves Avenue, Porto Alegre 91501-970, Brazil; weslei.ambros@ufrgs.br; 5 Departament d’Enginyeria Minera, Industrial i TIC, Universitat Politècnica de Catalunya, Barcelona Tech. Av. Bases de Manresa 61–63, Manresa, 08242 Barcelona, Spain

**Keywords:** construction and demolition waste (CDW), CDW recycling, construction materials from CDW, recycled concretes, CDW adsorbents

## Abstract

A literature review comprising 163 publications published over a period of 26 years from 1992 to 2018 is presented in this paper. This review discusses the generation and recycling of construction and demolition waste (CDW) as well as its main uses as raw materials for the construction engineering sector. This review pays attention to the use of CDW aggregates for sand, pavements/roads, bricks, ceramics, cementitious materials, and concrete productions, as well its uses as eco-friendly materials for water decontamination. The physical-chemical and mechanical characteristics of recycled aggregates play an important role in their correctly chosen applications. The results found in this literature survey allow us to conclude that recycled aggregates from CDW can be successfully used to produce construction materials with quality comparable to those produced with natural aggregates. We concluded that the use of CDWs as raw materials for manufacturing new construction materials is technically feasible, economical, and constitutes an environmentally friendly approach for a future construction and demolition waste management strategy.

## 1. Introduction

Waste management is one of the most challenging problems of the 21st century. Among the main types of wastes, construction and demolition waste (CDW) has received important attention in the waste concern [1,2]. CDW is one of the heaviest and most voluminous waste streams generated in the European Union and across the world. CDW is usually defined as the waste materials from operations of construction, reconstruction, alteration, extension, maintenance, and demolition of buildings and other infrastructure [3].

The construction and demolition industries generate around 900 million tons of waste per year, in the European Union alone [3]. CDW is produced in virtually all activities related to the construction and demolition sectors, such as the construction of buildings, houses, roads, bridges, flyovers, etc. These residues consist of distinct types of materials and are a heterogeneous residue that can contain any constituent that is part of a building or infrastructure, as well as any other materials used during construction work [1,4]. In this way, it is comprised mostly of inert and non-biodegradable material such as sand, gravel, concrete, metal, plastic, glass, etc. Therefore, inert fraction waste represents between 40 and 85% of the overall waste volume, discounting excavation soils from this waste [3].

**Figure 1:**
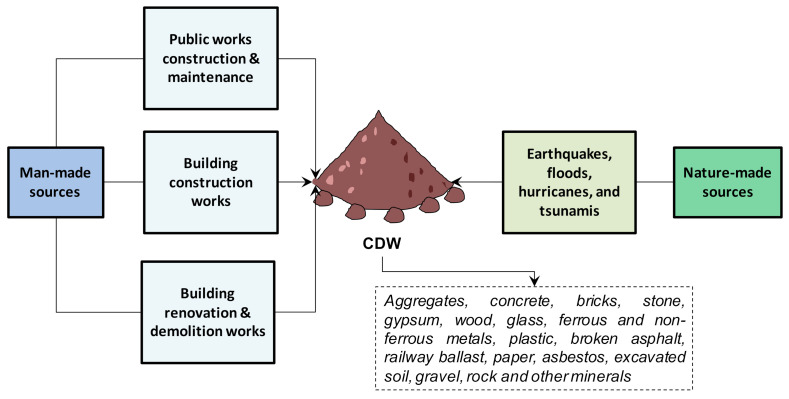
Classification of construction and demolition waste (CDW) according to the source of origin. Adapted from Menegaki and Damigos [1].

Figure 1 shows a generic schema of CDW classification based on its sources of origins [1]. It is well known that the amount and composition of any residue, and in particular, CDW, can vary widely according to the regions in which they are generated, mainly depending on factors such as population growth, regional planning, legislation, soil characteristics, topography, construction materials, and technologies, etc. [1,3].

The CDW amount is expected to increase in Europe every year; therefore, within this scenario, new strategies for its reuse and recycling need to be implemented correspondingly. Table 1 shows the CDW quantities produced in the E.U. and other countries in the world, as well their recovery/recycling rates indexes. According to the E.U. Waste Framework Directive (2008/98/EC) [5], member states were required to set a target for the recycling of non-hazardous CDW at a minimum of 70% of its weight by 2020. According to the final report published by European Union [2], it showed that fourteen member states already achieved the 70% target (see Table 1), and six other countries were very close, with a recovery rate higher than 60%. On the other hand, some member states showed very low recycling rates, such as Greece, Finland, and Bulgaria [2].

The potential for the recycling and reuse of CDW is high. There is a huge market interest to reuse and recycle aggregates derived from CDW in construction materials and projects [6,7,8,9,10,11,12,13,14,15,16,17]. Over the last decades, many studies have been performed to evaluate the feasibility of using CDW in projects, such as:

✓ Concrete, mortar, and ceramic materials [4,7];
✓ Eco-friendly concrete blocks [8,10];
✓ Geopolymers synthesis [11,13];
✓ Geotechnical applications [14];
✓ Use in sea-wall foundations [15];
✓ Landfill cover layer [16];
✓ Alternative pipe backfilling materials [17];
✓ Asphalt and roads [18,19,20].

Within the aforementioned context, the main goal of this review is to fill the information gap regarding the use of CDW in the production of construction materials, particularly concrete blocks, concrete, cement, ceramic bricks, and roads, as well their uses for environmental applications. Special attention is given to the quantification and properties of CDW generated in Europe and worldwide. Moreover, we hope that the current study may help to promote the good management of CDW, which is still a challenge to the global construction industry. Open atmospheres about the treatment and utilization of CDW should be built in the international industrial community.

## 2. Composition and Properties of Recycled Materials

### 2.1. Composition and Chemical Characteristics of Recycled Aggregates (RAs)

Recycled aggregates (RA) composition is mainly dependent on its sources (concrete, masonry, bricks, roads, etc.), which can vary by location. In some countries, fired bricks are the main construction material, while in others, masonry and wood are the main materials [3]. Building and construction technology has a large influence on the chemical composition of CDW. For instance, the presence of sulfate from interior rendering or glass from glazed ceramic tiles can seriously affect the use of RA produced from contaminated CDW. 

**Figure 2:**
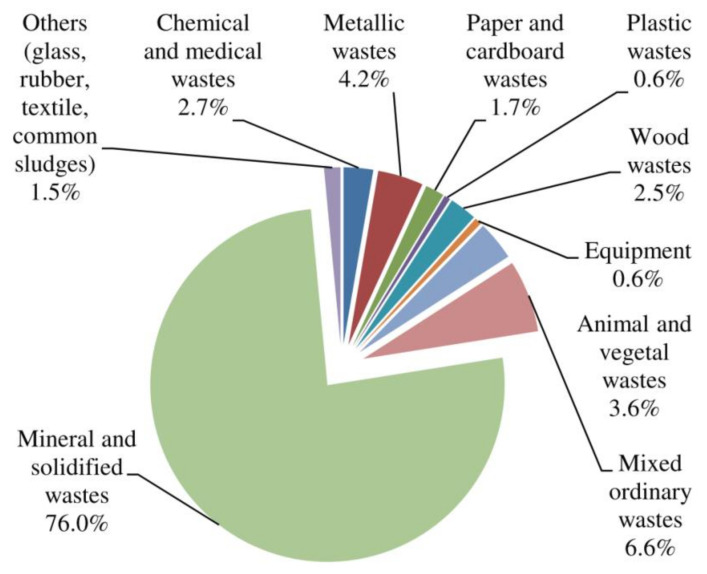
Total waste generated in the European Union according to the waste category, 2017 [3,21].

Figure 2 shows the general characteristics and composition of the solid wastes generated in Europe according to the waste category. It shows that the fraction of the mineral and solidified wastes (concrete, masonry, bricks, ceramics, earth, etc.) are very high, reaching up to 76%, and coming mainly from construction and demolition activities. However, many other types of wastes are generated and sometimes mixed with CDW. This can affect the composition of CDW and the recycled aggregates (RAs) from CDW, which also affect its reuse and recycling, which will be further discussed in the paper. An example is given in Table 2, which indicates that the general composition of CDW aggregates (Recycled Ceramics (RC) siliceous and limestone and recycled mixed aggregates (RMA) and recycled mixed ceramic aggregates (RMCA)) varies in terms of its main oxides. The aggregates’ compositions largely depend on the nature of the aggregates used in their manufacture, such as type of cement, sand, or siliceous aggregates.

### 2.2. Water Absorption

Water absorption (WA) is a key factor affecting the durability of a material and its resistance to the natural environment. The durability of construction materials is mainly dependent on permeability, which is the capacity of a fluid to penetrate their structures. High permeability leads to the penetration of water and ions molecules that react with, i.e., harden, cement paste microstructures, destroying their chemical stability [23]. On the other hand, and considering the service-life of construction, low permeability improves the resistance of the construction materials because it can avoid or impede the degradation agent attacks [23,24].

Natural aggregates have WA values between 0.5% and 1.5%, which is normally omitted for most concrete applications. However, more precautions must be taken when using RAs because their greater porosity can lead to WA values up to 12%, which therefore might lead to mixtures containing 1000 kg of RA in 1 m^3^ of concrete easily containing 100 liters of absorbed water [25,26].

The high WA of RA is related to the presence of old mortar. The ability of a recycled aggregate to absorb more water is due to the high presence of the adhered mortar onto the aggregate’s surface which, consequently, leads to a higher porosity of aggregate; this can affect the amount of water available for mixing, also influencing the cement hydration and therefore leading to problems related to the loss of concrete workability at the fresh state and the loss of mechanical and durability performances in the long-term [27]. The high presence of attached mortar enhances the porosity and WA of the aggregate, which is responsible for the concrete density and durability reduction. RAs have higher WA compared to natural aggregates (NA); therefore, there is also a critical disadvantage of reusing it in new construction materials, which is the heterogeneous nature of RA [28]. Although NA generally comes from the same resource, RA has several origins and qualities, leading to quality variation between different batches [28].

Peng et al. [29] performed an experiment evaluating the recycled concrete properties made with RAC. The WA of RCA was much higher than that of NA. The WA of RCA decreased from 4.07% to 2.89% and from 7.89% to 2.77%, respectively, after removing the attached mortar. Additionally, the authors stated that the microcracking of original gravel in RCA is also responsible for increasing the WA.

Taking into consideration the cementitious aggregates, its fine fractions contain higher cement mortar amounts than coarse fractions, which leads to much higher WA. If used in new concretes, high WA will affect the water/cement ratio, workability of concrete mix, and physical properties such as water absorption and carbonation. Therefore, it is important to remove the old mortar adhered in the RA that will be used in the new cementitious material.

### 2.3. Density

As well as the water absorption, the density of an aggregate is an important property. It defines the quality of the material and is important for proportioning concrete mixes and controlling several properties of the hardened recycled aggregate concrete [30]. The recycled concrete aggregate’s density is proved to be lower than that of natural aggregate. This is due to the presence of adhered cement mortar [31] and peculiar characteristic of the recycled concrete aggregates with respect to natural ones. This adhered material makes the aggregate density lower: typical values range between 2.2 g/cm^3^ and 2.6 g/cm^3^ [32].

De Juan et al. [33] showed the influence of the amount of adhered mortar on recycled concrete aggregate density. It was shown that depending on the liberation degree, density can vary along with the different grain size fractions, i.e., fine fraction, where the old cement paste is concentrated, it can show a different density of coarse fraction [34].

The strength of the original crushed concrete only marginally influences the density of the resulting aggregate [35,36,37]. Topçu and Sengel [38] evaluated the influence of the addition of recycled aggregates in new concrete production. The fresh-state results showed, as expected, a decrease in the density and workability caused by the replacement of the NA by RA. Limbachiya et al. [39] found that the variation of the particle density of the RA was 7–9% lower than that of the NA, which resulted in recycled concrete with worse mechanical properties.

When the material is composed of crushed concrete and masonry rubble, the density values are lower than for recycled concrete aggregate [40]. Red ceramics density is not only lower than concrete density [41,42,43], but also varies with the manufactured temperatures [44]. In this context, homogenization of the feed mixed recycled aggregate [45] becomes of major importance in producing secondary raw materials.

Mortar, of which density is generally lower than 2.1 g/cm^3^, is another RA component that could drastically influence density, but its content is generally limited by the RA producer, as is the case for other impurities such as wood, gypsum, glass.

### 2.4. Environmental Limitations of the Use of CDW in Relation to Its Constituents

Ascertaining the physical and chemical composition of RAs is important, because based on these data, future approaches for their reuse will be evaluated and proposed. Some factors determine the potential reuse and recyclability of CDW into RA for any specific situation. CDW can contain high concentrations of harmful substances that sometimes exceed regulatory limits and therefore they can be environmentally incompatible to be used as RA. In that case, they could require appropriate disposal or segregation by a separation technology able to remove/reduce the amount of such contaminants.

If it is not properly managed, CDW might contain small amounts of several hazardous substances such as metal-based paints, adhesives, phenols, resins, aromatic hydrocarbons, and others [21]. Once these substances are in the CDW and those are used as RAs for construction materials such as concrete, bricks, and roads, they can pose particular risks to the environment by contact with rainwater or infiltration, which can leach out these harmful elements (including organic compounds, anions, and metals). Such leaching represents a potential risk to the environment.

Leaching tests on construction materials containing waste [46,47] are being conducted in view to better understand the effect of incorporating recycled aggregates in concrete, especially on the increase in metal trace element (Cr, Pb, As, Ba, Cu, Mo, Sb, Ni, and Zn) and anion (sulfates and chlorides) amounts, and consequently, their potential release.

Engelsen and co-workers [48] showed that when the value pH was high, the amount of metal trace element leaching was low because of the interactions with hydrate constituents. Oxyanion-forming elements could be integrated into the structure of the hydrated phases, ettringite or hydrocalumite, hence limiting the leaching process [49]. After modelling the leaching behavior, Engelsen et al. (2010) [50] concluded that there was no difference between the leaching behavior of classical and recycled concrete.

### 2.5. Limitations on CDW Composition in Relation to Its Applications

The variety of contaminants that can be found in RA from CDW might also put structures at risk because many of these elements can severely degrade the strength of concrete made with RA from CDW. Such materials include asphalt, glass, gypsum, metals, plastic, rubber, soil, or wood. For instance, Hansen [51] reported that the addition of 30% by volume of asphalt in recycled concretes can reduce its compressive strength by about 30%. Huang [52] reported that 75% compressive strength of recycled concrete was lost with a replacement level of 64% of asphalt aggregates, by weight of total aggregate content. According to the European Standard EN 12,620 [53], the maximum allowed bituminous material content in RA is about 10%.

Other constituents such as glass are commonly found in CDW, even though it is usually removed from buildings and houses before demolition. Organic materials, for example, wood and plastic, are also often found in CDW and can be removed through density separation techniques [54]. It is known that the presence of these constituents in CDW provokes a very harmful effect on the qualities of the construction materials concerning their mechanical properties; therefore, their separation is needed. According to the European Standard EN 12,620 [53], the glass content must not exceed 1% by mass, and wood and plastic, which float in water, are classified separately as floating non-stone material; content is limited to a maximum of 0.1% by mass [21].

Ascertaining the correct quantification of constituents in CDWs is important for understanding their chemical compositions, and therefore their possible applications or processing needs. Considering the vast range of environments and conditions to which these materials made from CDW can be exposed, their chemical composition (e.g., sulfate and chloride content) could compromise the performance of recycled concrete, bricks, and roads made from CDW.

For instance, in road aggregates, the amount of sulfur compounds is very limited to ensure its chemical stability and avoid pathologies in adjacent structures of concretes or pavement layers made with cement. Gypsum is the main source of soluble sulfates in RA, and there is a linear relationship between the total sulfur content measured according to EN 1744-1 [55].

Agrela et al. [56] studied the soluble sulfate (SO_3_) in natural aggregate and mixes of CDW (crushed concrete and crushed masonry). They found values of 0.05% for NA and values from 0.69 to 0.72 in different mixes of CDW; these values are below the limit for soluble sulfate, and the total sulfate content must not exceed the specified limit of 1% for pavement structural layer materials according to UNE EN 103,201 and EN 1744-1 specifications [57]. Agrela et al. [58] found total sulfur values from 0.37 to 1.58 and soluble sulfates varying from 0.22 to 1.09%. Vegas et al. (2011) studied CDW aggregates for roads and showed that those aggregates which presented soluble sulfate contents below 3.74% did not cause dimensional stability problems for the roads.

However, in the presence of easily mobilizable sulfates, an internal sulfate attack manifested by a delayed swelling of hydraulic mixtures may happen. This is due to the formation of a significant amount of secondary or delayed ettringite [59]. It appears that this reaction requires simultaneous specific conditions such as a moist environment, high temperature of the concrete at early age, and presence of aluminates.

Recycled aggregate could also contain significant quantities of soluble alkalis and potentially reactive silica and may present a risk for alkali–silica reactions (ASRs) [60,61] when used for producing new concrete. This chemical mechanism [62,63] can induce cracking and severe damage in concrete structures [64,65,66]. Methods have been proposed in order to limit the risk of ASRs when using natural aggregates [67,68], although their adaptability to RA has not yet been clarified.

## 3. Recycled CDW Materials Applications

### 3.1. Sand Production

In the construction sector, river sand is the most commonly used aggregate in cementitious materials. On one hand, there is a worldwide shortage of good-quality natural sand, whereas in countries where the availability of sand is sufficient, the mining costs and environmental impacts related to extraction, processing, and transport are other major concerns with the ever-growing usage of this natural resource [69].

Based on cement usage and engineering computations (i.e., cement consumption and the ratio of cement to sand for various construction purposes), sand demand for 2007 was estimated to be 17.37 million cubic meters, and since then its usage has grown [70]. Additionally, the last UNEP (United Nations Environmental Program) report “*Sand and sustainability: Finding new solutions for environmental governance of global sand resources*” [71] estimated a sand demand of about 50 billion tons per year, an average of 18 kg per person per day.

Along with these issues, the European Parliament (according to Waste Framework Directive 2008/98/EC) stated that EU countries should achieve 70% of CDW minimum recycling rate by 2020. In this regard, over the last few years, studies have been carried out into using CDW materials as a replacement for sand in concrete materials.

Silva et al. [72] studied the addition of fine crushed red clay brick to a siliceous sand mortar. Three different ratios were tested (0%, 5% and 10%). In general, the authors reported that the addition of red clay brick waste up to 10% improved the mortar properties in comparison with the reference mortar.

In another study [73], a comparative analysis of mortars prepared with three natural sands and three recycled sands was performed. The properties of these mortars were fully evaluated and compared. The shrinkage and water absorption were higher in mortars with recycled sands, while compressive strength was lower when compared to reference mortars.

From an environmental perspective, the use of fine RA as sand can bring several advantages such as: (i) it reduces the sand mining, which it causes huge environmental impacts worldwide; (ii) it reduces the consumption of energy and CO_2_ emissions; and (iii) it prevents illegal deposits and landfill of the fine fractions of CDW.

However, the main issue concerning the quality of recycled aggregates from CDW for sand production is the presence of the porous and low strength phases (adhered mortar), and specifically to the patches of hardening cement paste attached to the surface of natural aggregates. This produces sand with low quality which, when used in cementitious materials (concrete or mortar), harms workability and the mechanical and durability performance at hardened state [74]. For those reasons, in most countries, recycled sands (made from CDW) are not allowed in structural cementitious material production.

Therefore, the removal of adhered mortar is a crucial factor for the improvement of aggregate performances; however, this is not a simple task. Thus far, it is seen in the literature that for the separation of cement paste from CDW successive comminution stages [75] and thermal treatments [76] are necessary which, due to their costs, are not generally employed, and in some cases increase the environmental impact of the final product [77].

### 3.2. Use of CDW for Pavements/Roads Construction

Pavements are one of the most energy-intensive infrastructure assets depending on non-renewable natural resources. Therefore, the application of recycled aggregates from CDW in the construction of embankments, sub-bases, and foundations for roads, where unbound materials are used, is an excellent management idea for increasing recycling rates and creating a market opportunity for recycled aggregates.

Currently, the main utilization of CDW aggregates is for pavement construction. Recycled aggregates from construction and demolition waste have often been used in pavement layers, from small percentages to the full replacement of materials [4,57,78]. In several countries, management and experimental testing technologies containing information about executive procedures and their performance have been gaining ground in the discussion of the environmentally correct reuse of materials.

In European countries and Japan, there are elaborate and consolidated policies regarding the control and management of waste due to the high demographic density and little storage space [4]. That is why these countries have pioneered the development of knowledge about how to handle CDW. Some countries in the world, such as Italy, have specifications for the control of production and application of recycled aggregates for paving, considering the shape and heterogeneity of the grains. Ekanayake and Ofori [79] reported that, in the United States, more than 20 individual states use CDW as aggregate in highway construction. In Brazil, The Netherlands, and Japan the recycling of CDW materials in paving and road works is mandatory, and a huge amount of CDW is used for this purpose [1,80].

Leite et al. [78] evaluated the feasibility of using RA for pavement applications. The behavior of the recycled materials was compared with the behavior of limestone aggregates. The authors concluded that RA from CDW, which has higher densities, might be utilized as a coarse base and sub-base layer for low-volume roads. In addition, RA rich in cementitious materials also helps to improve the layer performances.

Molenaar and van Niekerk [81] evaluated the effect of the composition, degree of compaction, and grading on the mechanical characteristics of crushed concrete and crushed masonry on unbound base. The authors found that all three factors influence the mechanical characteristics of pavement, but it was the degree of compaction that affected it the most. In practice, this is an interesting outcome, because the degree of compaction is much easier to control than other factors such as gradation and composition. In another study [82], the performance of RCA in the base and sub-base was studied. The results showed that a mixture of 25% RCA with 75% NA obtained the same resilient response and permanent deformation properties as a dense-graded coarse aggregate base, which is used in base and sub-base layers.

Although the use of CDW for road construction has been widely performed, the standardization of the RA used in roads is currently under way. Most countries are implementing standardized limits on the use of NA in roads. For instance, the presence of sulfur compounds is a limiting factor, not only for concrete but also in road aggregates, to ensure the dimensional stability of the section and avoid potential pathologies in adjacent concrete structures or cement-treated pavement layers [83].

Agrela et al. [56] studied the soluble sulfate (SO_3_) in natural aggregate and mixtures of CDW (crushed concrete and crushed masonry). They found values of 0.05% for NA and values from 0.69 to 0.72% in different mixes of CDW; these values are below the 1% limit for soluble sulfate which the total sulfate content must not exceed for materials used as pavement structural layer according to UNE EN 103,201 and EN 1744-1 standards [57]. The recycled aggregates used in the rural road met the limit of 1% for sulfur compounds set by the technical specification. However, Vegas et al. [84] have questioned this limit. Vegas et al. [85] studied CDW aggregates for roads and showed that these aggregates which presented soluble sulfates content below 3.74% did not cause dimensional stability problems for the roads. Many other parameters including maximum aggregate size, particle shape, grading (especially fines content), density, etc., have been identified as affecting the road performance [4]. 

Another important method of CDW reutilization is to use reclaimed asphalt pavement (RAP) in new roads and or pavements. RAP has become a reality in many countries such as the United States, China, Egypt, Japan, and Australia, among others [86,87]. This practice is becoming popular due to both environmental and economical attractiveness. For instance, Vidal et al. [88] showed that the incorporation of 15% RAP into new asphalt mixtures could reduce the total cumulative energy requirement, climate change, and use of fossil fuels by 13 to 14%. Edil [89] reported that up to 30% of savings could be achieved by using RAP as a base material for pavements. Asphalt binder is the most expensive material; therefore, using RAP material in pavements it means that less binder is required.

Mousa et al. [87] studied the application of RAP in the construction of unbound base and sub-base layers in Egypt. The authors studied what the optimal amount of RAP (0%, 20%, 60%, 80% and 100%) was which could be blended with NA. It was concluded that mixing up to 60% of RAP in road sub-base and 20% in road base construction could be used. Much other research has been conducted in this regard.

In another investigation, extensive research was performed in Florida, U.S.A. [90], related to the use of RAP to build sub-base layers below rigid pavements. The RAP layer performances were evaluated and compared (over one year) to a lime rock control section. The authors stated that the mechanical performance of a sub-base layer constructed with RAP was similar or even better than the one constructed with lime rock. Moreover, it was concluded that no environmental impact was detected when RAP was used as a pavement material for highways.

### 3.3. Ready Mix Concrete

Concrete made with RA is no longer only a research field and, in many countries, it is a practical reality [91,92]. Various pilot projects have been implemented in countries such as China, the United States, Portugal, Germany, France, and Brazil with encouraging results [43,57,59]. Such use is becoming so widespread that several countries have developed or are developing normative documents to address the specificities of using recycled aggregates for concrete [93,94]. This conveys the significance of studies researching the suitability and performance levels of these residues in high-level recycled applications, i.e., structural concrete.

Over the last few years, CDW has been extensively studied for recycling and the application as aggregates for producing new concrete [74,92,94,95,96]. However, despite many types of research involving CDW for concrete production, there is still a lack of confidence in the construction sector about using the RA in real concrete applications because of its “poor” mechanical performance compared with natural aggregates.

Concrete is composed of three components: aggregate, hardened cement paste, and an interfacial transition zone (ITZ) between the cement paste and the aggregate [93]. Usually, the ITZ is the weakest part because of its higher porosity and cracks than in the other components [97,98]. Concrete made with RCA results in more ITZs than in concrete made with NA. Shi et al. [93] found that the adhered original mortar was the weakest portion in recycled concrete. The presence of the residual mortar in RA can vary up to 60% depending on the aggregate grain size. Indeed, the most distinguishing feature of RA is the presence of old adhered mortar, which makes it significantly porous due to the high porosity of hardened cement paste, resulting in an inhomogeneous and less dense aggregate, and, consequently, in a poor quality of the concrete.

Butler et al. [97] indicated that RCA with good quality should meet certain criteria to be suitable for use in reinforced concrete. The relative density of the aggregate should be 2.3 or higher, and a maximum mortar content and water absorption of 50% and 3%, respectively. This limit in water absorption is hard to follow in practice, and the effects of RA in concrete appear both at fresh and hard states. The higher water absorption is responsible for difficulties in maintaining workability, especially during transportation [99] and casting [100]. At hard state, the water absorption and porosity of RA affect concrete characteristics in rather complex manners. For instance, the ITZ microstructure of recycled aggregate concrete can be strengthened by carbonation treatment of recycled concrete aggregate [101]. The adhered cement paste of the RAs can generate a higher creep or drying shrinkage in concrete [102,103]. Additionally, although the durability could be affected [104], the RA excess water could act by internally curing the concrete [105,106].

Another aspect that should be addressed is the risk of contamination of using CDW aggregates. Because the RCA comes from debris of concrete elements, the risk that this material be contaminated with chloride exists. Studies showed the presence of chloride in recycled aggregates but always below the limits specified by standards [107,108]. With regard to the effect of RCAs on the chloride penetration resistance of RAC, published results showed that the RCA content affects the chloride penetration resistant. For a 100% replacement of coarse natural aggregates by recycled ones, the chloride penetration resistance can double the value of the correspondent reference concrete with natural aggregates [74,109,110,111]. Chloride resistance can be improved by reducing the permeability, i.e., reduction in porosity.

An alkali–silica reaction (ASR) is a harmful reaction generating expansion, cracking, and damages to concrete structures [112]. The incorporation of RCAs coming from concrete debris affected by ASR can initiate the expansion reaction in new RAC. The magnitude of the expansion is usually the same as the original concrete but can be larger due to the higher water absorption of the RCAs [113,114,115].

### 3.4. Concrete Blocks

Concrete blocks (CBs) are one of the main building materials used in the construction industry, and they are made using cement as a binder. A concrete block is one of several precast concrete products used in construction. It is made from a mixture of powdered Portland cement, sand, gravel, and water, and is responsible for high energy expenditure and a large carbon footprint [116,117]. Therefore, aiming for more environmental protection and sustainable development, several studies have been carried out on the production of CB from waste materials, in particular CDW aggregates, which are interesting materials for CB production [116,118].

Re-utilization of inert CDW as an additive for manufacturing concrete blocks is a winning strategy because it not only recycles the waste product, but also reduces the pressure concerning waste disposal as well as overcoming the shortages of clay in many parts of the world. Moreover, recycling CDW by incorporating them into CB for building materials is a practical solution for the pollution problem and reduces costs in the building sector. Therefore, the use of CDW waste as aggregates for concrete block production has attracted much interest in recent years, and few reports have been published dealing with the use of CDW as the main additive for fired brick production.

Besides the characteristics of the materials, the quality of the CB also depends on the fabrication method, drying and curing procedure, firing procedure, etc. [117]. These factors will affect the quality of the final product properties such as compressive strength, water absorption, impact and abrasion, low tensile strength, etc. Good quality CB has high compressive strength and low water absorption. Compressive strength is highly affected by firing temperature, method of production, and physical, chemical, and mineralogical properties of the raw material [117,119]. CB made of CDW with different compositions can also present different properties.

Poon et al. (2002) [12] produced CB by using cementing recycled aggregates and fly ash as main additives and found that replacing NA with RA at levels of 25% and 50% exhibited marginal impacts on the CB compressive strength. In relation to the compressive strength of the CB, some studies have reported that there was a very low effect when CB contains up to 50% of RCA in its composition [12,117]. Moreover, research has demonstrated that RCA with a higher cement content is needed for it to be a good additive to make CB with higher compressive strength [117]. In addition, regarding RA and RCA replacement ratios in CB, these wastes can be used in large quantities (up to 100%) because of their properties such as high hardness, high strength, chemical inertness, etc. Besides, these wastes are among the most popular recycled materials used in CB and could reduce the demand for NA in the CB industry.

The production of CB by firing is also an interesting alternative. Kou et al. [119] explored the feasibility of using fresh concrete waste for manufacturing wall blocks under different temperatures (300, 500, 800 °C). All the blocks were burnt after 28 days of curing. It was concluded that the compressive strength of all manufactured blocks increased when exposed to 300 °C, which was due to the acceleration of cement hydration. However, as the fire temperature rose (up to 800 °C), the compressive strength of the wall blocks significantly decreased. This happened because at high temperatures, the hydrated cement paste might be disintegrated (e.g., the hydration products such as Ca(OH)_2_ are decomposed at 500 °C and calcium silicate hydrates are decomposed at 800 °C) [12].

The production of concrete blocks by firing has the advantage of easy execution by using well-known processes. However, it has the disadvantages of consuming a significant amount of energy and releasing a large amount of greenhouse gases. Despite many studies which have been carried out for producing bricks from RA, the commercial production of bricks from waste materials (specially from CDW) is still very limited. Some drawbacks related to brick production include the absence of relevant standards, the potential contamination from the waste materials used, and the slow acceptance of waste material-based bricks by industry and the public [117]. Therefore, boosting the production and utilization of CBs made from CDW materials requires studies not only on the technical, economic, and environmental aspects, but also on standardization, government policy, and public education [117].

### 3.5. Cement

Cement an essential material for the economic development of cities because it is the most consumed material by construction industries. However, its production is extremely energy-intensive and leads to excessive pollution including SO_2_ and CO_2_ emissions. According to the Cement Technology Roadmap [120], the UNEP report on low-CO_2_ eco-efficient cement-based materials [121,122], the cement industry contributes to about 5–8% of the global CO_2_ emissions, which are mainly released from the calcination of limestone; therefore, it is one of the most impacting sectors to be duly considered in “green strategies”.

In 2010, 3.3 billion tons of cement were produced worldwide, with an increase of 7% in the preceding year [123], reaching 4.13 billion tons, in 2016 [124]. In 2020, the cement production increased to 4.13 billion of tons, and a production of 4.83 billion of tons is expected by 2030 [124]. However, considering the infrastructure development in Asia and other emerging economies, such as Turkey and Brazil, cement production will further increase. Therefore, efforts have been made to produce environmentally friendly cementitious materials from waste materials [125].

The main raw materials for Portland cement production include limestone (CaCO_3_); clay, as a source of alumina (Al_2_O_3_); silica (SiO_2_); and ferrous oxide (Fe_2_O_3_). Therefore, theoretically, any material which has all these oxides can be used as a raw material to produce Portland clinker. This is the case with CDW, which is mainly composed of these minerals. These can also include recycled concrete aggregates, especially the fine fraction rich in old, hydrated cement paste [126] and other waste coming from cementitious products [127].

Another field of application of RA is the alkali-activated cement, so-called geopolymers. Allahverdi and Kani [128] developed a geopolymer using waste from a brick production plant and activated it with NaOH in a proportion of 8% Na_2_O in the binder. This cement had a compressive strength of 40 MPa after 28 days of curing. In another study, Allahverdi and Kani [128] reported compressive strengths of up to 50 MPa using a mixture of 60% concrete waste and 40% red clay brick waste activated with a solution of NaOH and water glass.

Puertas et al. [129] produced cementitious materials by using ceramic waste made from red and white clays, where the residue was activated with NaOH and water glass. The authors found that the samples cured for eight days exhibited compressive and flexural strengths up to 13 MPa. Komnitsas et al. [11] evaluated using various construction residues including bricks, tiles, and concrete for manufacturing geopolymer cement, and reported compressive strengths of up to 57.8 MPa. In another published work, geopolymer cement was manufactured by using ceramic wastes, and after 28 days of curing, the specimens exhibited a compressive strength up to 71 MPa [130].

Impurities and contaminants that can be found in CDW are one of the main issues for cement production because they can affect its properties. According to Duxson et al. [131] and Luukkonen et al. [132], the content of SiO_2_ and Al_2_O_3_ plays an important role in the performance of cementitious materials. According to the authors, increasing the SiO_2_/Al2O_3_ molar ratio increases the compressive strength and elasticity up to a certain ratio. Porosity, in contrast, increases at low SiO_2_/Al_2_O_3_ ratios. Hajimohammadi and van Deventer [133] reported an opposite trend; a SiO_2_/Al_2_O_3_ ratio of 1.8 resulted in a higher compressive strength than SiO_2_/Al_2_O_3_ of 2.25. These studies show that the trend of compressive strength as a function of the composition (in terms of SiO_2_/Al_2_O_3_ ratio) is not constant across materials and depends on other additional factors.

Despite the important achievements of the cement industry, the technology of cement production will, without doubt, be developed further in future. However, it will be necessary to overcome certain challenges which remain, such as reducing production costs and keeping a strong focus on quality, performance, influence of cement on concrete durability, and the decrease in CO_2_ emissions related to its production.

### 3.6. Ceramics and Bricks

The utilization of solid wastes as additives to manufacture ceramic bricks and products has attracted huge attention over the last few years. It is interesting to both the enterprises generating wastes and the producers of ceramic bricks, stones, tiles, etc. Ceramic materials show an extensive range of chemical composition, resulting in products with heterogeneous characteristics [134]. This attribute enables easy incorporation of different types of waste materials into its products, which is the case of the inert CDW [117].

In general, inert CDW minerals are very heterogeneous but contain almost always the same main components as those of natural ceramic raw materials such as mortar, ceramics, concrete, rocks, natural gravel, masonry, sand, and soil, depending on the place they are generated and characteristics of each construction [134]. These characteristics might qualify it as a good additive for ceramic material production. Thus far, very few studies have dealt with the use of CDW as an additive for ceramic material construction [135,136,137,138].

Acchar and coworkers [139] investigated the effects of the incorporation of CDW on the properties of clay-based ceramics. The results demonstrated that a high content (approximately 50 wt.%) of CDW can be added into red tiles and bricks ceramics without causing changes in the processing routine and without causing harm to their characteristics.

Gaspareto and Texeira [140] proposed to produce ceramic bricks by using crushed CDW as a de-plasticizing material and mixing it with natural clay with high plasticity, to adjust the properties of the fresh mixture. The physical properties of the ceramic mass were evaluated after burning, aiming for its application in the production of solid bricks. The results indicate that it is possible to use CDW with this clay to produce massive ceramic bricks, obtaining a ceramic material with good physical properties. In the tests of compressive strength, it was possible to conclude that adding 40% (wt.%) had a compressive strength higher than 4 MPa, considered very good for the Brazilian standards.

Bianchini et al. [135] also studied the effect of the incorporation of CDW on the sintering/densification and mechanical behavior of a commercial clay-based ceramic mixture for ceramic brick production. The obtained results showed that high contents (approximately 20 wt.%) of CDW can be incorporated into an industrial clay mixture for ceramic products without significant changes in its properties.

Dos Reis et al. [141] prepared fired bricks by using sludge from the inert mineral part of the construction and demolition waste with different proportions (0%, 30%, 50%, 70%, and 100% by weight). The results showed that this waste can successfully be used as the main additive for brick production. The brick made with 70% of the waste presented the highest compressive strength value (16.8 MPa) in comparison with the other proportions. Furthermore, the addition of the waste improved the insulation properties compared to the clay brick.

However, some concerns need to be addressed before inert CDW or any waste can be incorporated as an additive in ceramic products; such as, the chemical composition and size and shape of the additives. For example, the presence of Fe_2_O_3_ can lead to problems of efflorescence when the clay is homogenized for a long time, and thus it is recommended not to exceed 10% of Fe_2_O_3_. The presence of CaO can also be a problem. During firing, CaCO_3_ is broken down, producing CO_2_ and CaO, and if free CaO does not bond, it can produce expansion in bricks by moisture absorption, and due to this, cracks or chipping may be produced [117].

The presence of carbon is also an issue of concern. When the carbon is not completely burned out during the firing of the ceramic products, it is coked inside the samples and that can lead to poor strength of the ceramic product [84]. The size and shape of the materials used as additives can also play an important role in the quality of ceramic products because such characteristics of additives may lead to a bad and/or cold compaction and thus to the low physical quality of the ceramic products [117,134]. Additionally, large grains are subjected to stresses beyond their limits and fracture, making the rest of the structure less resistant.

### 3.7. Environmental Application for CDW—Adsorbent Material to Clean Up Polluted Waters

As mentioned before, there are many possibilities for CDW recycling and reuse, most of them related to the use of CDW in construction materials. However, the uses of CDW as raw materials for environmental applications as eco-friendly materials have grown significantly, such as its use as an adsorbent for the uptake of pollutants in aqueous media [141,142,143,144,145,146]. A compilation of cementitious adsorbents derived from CDW and their conditions of application for pollution removal from aqueous media is shown in Table 3.

Adsorption processes represent a cost-effective approach for solving many problems of the treatment of wastewaters [158,159]. Adsorption is a surface process where pollutants are transferred from the effluent to a solid phase. One advantage of adsorption technology is that the adsorbents can be regenerated and reutilized. This merit makes it a low-cost process, even cheaper when used with wastes such as CDW.

To date, some adsorbents made by CDW have been reported in the international literature (see Table 3). These adsorbents have been employed in the removal of several pollutants from aqueous solutions, such as heavy metals, arsenate, dyes, drugs, and fluoride, etc. The maximum adsorption capacity (Q_max_) was used to evaluate the effectiveness of these CDW adsorbents. From Table 3, it can be observed that the Q_max_ varies according to the pollutant used, as well as the characteristics of the adsorbents. Moreover, the adsorption studies have used different experimental conditions such as different initial concentrations of pollutants, varied contact time, temperatures, and pH conditions. It is well known that these conditions play a huge influence on the efficiency of uptake amount of the selected pollutant. However, the data show that even using different pollutants, different adsorption conditions and different CDW characteristics as adsorbents, they can be successfully employed in the adsorption process.

Kagne et al. [147] studied the removal of fluoride in synthetic and real wastewaters on hydrated cement and demonstrated that its uptake mechanism was due to chemisorption and precipitation. The same findings were found and related by Bibi et al. [160]. Sasaki et al. [143], using concrete wastes for the removal of arsenate, found that its removal mechanism involved the precipitation of arsenate in the form of calcium arsenate (Ca_3_(AsO_4_)_2_) and by ion exchange with ettringite present in the concrete matrix. Littler et al. [161] studied phosphate adsorption and have shown that, in the presence of cement (or similar), the dissolution of calcium into phosphate-bearing waters results in the precipitation of calcium phosphate solids. The same phenomenon was observed and related by Park et al. [162].

However, the main concern of using concrete materials as an adsorbent for effluent treatment is that they are not completely inert materials. Generally, they present potential hazardous elements such as heavy metals, basic elements, and other toxic constituents that can be leached into the soil, leading to environmental risks. Therefore, investigating metal speciation and leaching behavior is an important way to determine and minimize risks to the environment.

Barbudo et al. [163] evaluated the leaching potential of NA and RA from CDW and the results showed that neither of these aggregates released detectable quantities of heavy metals. However, a high concentration of SO_3_ compounds was detected which can cause the pollution of superficial and/or groundwater. Martemianov et al. [145] applied concrete adsorbent for removing metals and arsenic in water and the results showed that the leaching of arsenic, copper, and lead met the requirements of drinking water standards, but the leaching of cadmium was high compared to the other metals.

These results have demonstrated that CDW exhibits good adsorption capacities towards different types of pollutants. Additionally, these data strengthen the potential of CDW materials to be applied as adsorbents to diminish the level of pollutants from wastewaters.

## 4. Conclusions, Remarks and Future Trends

Environmental concerns about CDW generation and accumulation rise every year, which reinforces the need to reuse it as recycled aggregate for construction industries, because the sector has a great potential to absorb most of the CDW generation. In this context, this paper provides a thorough literature review on the current situation and challenges in the application of recycled aggregates from CDW considering a worldwide scenario. CDW generation, composition, properties, and alternative uses are reviewed in detail. Seven main applications for recycled aggregates obtained from CDW are presented and discussed: sand production, pavement/road construction, ready mix concrete, concrete blocks, cement, ceramics/bricks, and low-cost adsorbent for wastewater treatment. The data found in this literature survey indicate that recycled aggregates from CDW can be successfully used to produce construction materials with quality comparable to those produced with natural aggregates and constitute an environmentally friendly approach for a future construction and demolition waste management strategy.

Three main issues have been identified for future actions and studies oriented to the recycling of CDW in new construction materials: (i) the development of standardized tests to orient specific regulations for using RA from CDW in new materials; (ii) investigating the environmental risks associated with the use of RA from CDW as well as ways to potentialize its application in high added-value sectors, such as ceramics and pollutant adsorbents; (ii) looking deeper at aspects related to political strategies to boost the confidence and acceptance of materials derived from CDW by professionals and society. The more knowledge about the capabilities of using recycled aggregates, the better one can arrive at solutions to overcome the current challenges.

## Figures and Tables

**Table 1:** Total CDW generation and CDW recovery indexes, 2017 [1,2].

Countries	CDW Generation (10^6^ tons)	Recovery Rate (%)
Europe Union		
France	246.70	59.00
Germany	201.00	85.00
United Kingdom	100.23	91.00
Italy	39.00	97.00
Spain	27.70	68.00
The Netherlands	25.71	99.00
Finland	16.00	12.00
Czech Republic	13.80	60.00
Portugal	11.40	74.00
Austria	8.30	92.00
Sweden	7.70	79.00
Belgium	6.95	86.00
Poland	3.51	68.00
Ireland	3.31	74.00
Hungary	3.00	65.00
Denmark	2.89	87.00
Estonia	1.94	75.00
Bulgaria	1.54	12.00
Romania	1.33	67.00
Slovakia	0.80	39.00
Greece	0.81	0.40
Croatia	0.68	52.00
Luxembourg	0.58	99.00
Lithuania	0.56	87.00
Slovenia	0.53	91.00
Malta	0.52	19.00
Latvia	0.40	96.00
Cyprus	0.14	45.00
Other countries		
China	1020.00	40.00
India	530.00	n.a.
U.S.A.	519.00	48.00
Brazil	101.00	6.14
Japan	77.00	80.50
Taiwan	63.00	91.00
Hong Kong	24.30	28.00
Australia	19.50	62.20
Thailand	10.00	32.00
Switzerland	7.00	28.00
South Africa	4.70	16.00
Norway	1.30	67.30

**Table 2:** Chemical characterization of different recycled aggregates according to Jiménez [22]. RC: recycled ceramic aggregates; RMA: recycled mixed aggregates; RMCA: recycled mixed ceramic aggregates.

	RC—Siliceous	RC—Limestone	RMA and RMCA
SiO_2_	45–60%	4–5%	40–50%
Al_2_O_3_	15–20%	1–2%	6–8%
Fe_2_O_3_	2–5%	1–2%	2–4%
CaO	5–7%	52–54%	20–28%
MgO	0.5–1.5%	0.2–0.8%	0–1%

**Table 3:** Comparison of adsorption capacities of different cementitious adsorbents from CDW.

Adsorbent	Adsorbate	Q_max_ (mg g^−1^)	Isotherm Model	Conditions	Ref.
Cellular concrete-supported	Arsenic	16.0	Langmuir	0.2 g of adsorbent in 50 mL with initial concentration from 10 to100 mg L^−1^; pH from 6.5 to 7.2	Martemianov et al. [145]
Cellular concrete-supported	Copper	53.0	Langmuir	0.07 g of adsorbent in 100 mL of at 180 rpm; pH of 5.0 and equilibrium time of 120 min	Martemianov et al. [145]
Hydrated cement	Fluoride	2.7	Freundlich	Initial ion concentration of 15.8, pH of 6.7, adsorbent dosage of 10 g/L, shaking speed of 150 rpm, contact time of 24 h	Kagne et al. [147]
Recycled concrete	Phosphate	6.88	Langmuir	pH of 5.0; particle size 2–5 mm; 2.0 g of adsorbent in 100 mL of solution	Deng and Wheatley [148]
Aerated autoclaved light concrete	Arsenic(III)	15.5	Freundlich	Temperature of 24 °C, adsorbent dose of 1.0 g/L, contact time of 30 min, pH of 7.0	Mondal et al. [149]
Burnt Crushed Concrete Granules (700 C)	Phosphate	21.55	Langmuir	pH 7.0; Equilibrium time of 30 min; Adsorbent dosage of 5 g/L	Kang et al. [150]
Burnt Crushed Concrete Granules (900 C)	Phosphate	8.47	Langmuir	pH 7.0; Equilibrium time of 30 min; Adsorbent dosage of 5 g/L	Kang et al. [150]
Carbonated concrete	Phosphate	30.6	-	pH 12.4; 22 °C, Equilibrium time of 104 min; Adsorbent dosage of 5 g/L	Dos Reis et al. [146]
Non-carbonated concrete	Phosphate	47.6	-	pH 12.4; 22 °C, Equilibrium time of 72 min; Adsorbent dosage of 5 g/L	Dos Reis et al. [146]
CSW	Phosphate	24.04	Liu	pH 9.4; 22 °C, Equilibrium time of 212 min; Adsorbent dosage of 5 g/L	Dos Reis et al. [151]
CSW-C	Phosphate	57.64	Liu	pH 9.4; 22 °C, Equilibrium time of 136 min; Adsorbent dosage of 5 g/L	Dos Reis et al. [151]
Functionalized CDW	Ciprofloxacin	138	Liu	Temperature of 40 °C, adsorbent dose of 1.5 g/L, contact time of 70 min, pH = 7.0	Caicedo et al. [152]
Concrete sludge	Borate	50.0	-	Temperature of 25 °C, adsorbent dose of 1.5 g/L, contact time of 70 min, pH = 7.0	Sasaki et al. [153]
Portland Pozzolana Cement	Fluoride	0.25	-	Temperature of 40 °C, adsorbent dose of 50 g/L, contact time of 27 h, pH = 2.0	Shyamal and Ghosh [154]
Concrete powder	Cesium	96.97	Langmuir	Temperature of 21 °C, contact time of 8 min, pH = 12.0	Kang et al. [155]
Cement carbon composite	Methylene blue	9.6	Langmuir	Temperature of 30 °C, adsorbent dose of 1.0 g/L, contact time of 3 h	Manjunath et al. [156]
Cement carbon composite	Methyl orange	20.20	Langmuir	Temperature of 30 °C, adsorbent dose of 1.0 g/L, contact time of 3 h	Manjunath et al. [156]
Portland cement derived adsorbent	Copper	145.8	Langmuir	Temperature of 25 °C, adsorbent dose of 10.0 g/L, contact time of 3 h, pH = 5.0	Lim et al. [157]
Portland cement derived adsorbent	Cadmium	177.9	Langmuir	Temperature of 25 °C, adsorbent dose of 10.0 g/L, contact time of 3 h, pH = 5.0	Lim et al. [157]

## Data Availability

Data sharing not applicable.
